# Case Report: Primary Thoracic Low-Grade Fibromyxoid Sarcoma in a Young Girl Presenting With Mediastinal Mass Syndrome

**DOI:** 10.3389/fped.2022.885068

**Published:** 2022-06-17

**Authors:** Yat Chi Chan, Amanda N. C. Kan, Liz Y. P. Yuen, Innes Y. P. Wan, Kevin K. F. Fung, Yiu-fai Cheung, Karen K. Y. Leung, Dennis T. L. Ku, Anthony P. Y. Liu

**Affiliations:** ^1^LKS Faculty of Medicine, The University of Hong Kong, Kowloon, Hong Kong SAR, China; ^2^Department of Pathology, Hong Kong Children’s Hospital, Kowloon, Hong Kong SAR, China; ^3^Division of Genetic and Genomic Pathology, Department of Pathology, Hong Kong Children’s Hospital, Kowloon, Hong Kong SAR, China; ^4^Department of Surgery, Prince of Wales Hospital, Chinese University of Hong Kong, Hong Kong, Hong Kong SAR, China; ^5^Department of Radiology, Hong Kong Children’s Hospital, Kowloon, Hong Kong SAR, China; ^6^Department of Paediatrics and Adolescent Medicine, Hong Kong Children’s Hospital, Kowloon, Hong Kong SAR, China; ^7^Department of Paediatrics and Adolescent Medicine, LKS Faculty of Medicine, The University of Hong Kong, Kowloon, Hong Kong SAR, China

**Keywords:** low-grade fibromyxoid sarcoma, thoracic tumor, pediatrics, mediastinal syndrome, RNA-sequencing

## Abstract

Low-grade fibromyxoid sarcomas (LGFMSs) are typically adult-onset tumors that arise from the extremities. Here, we report an exceptional case of primary thoracic LGFMS in an 8-year-old girl that resulted in mediastinal syndrome. In reporting this case, we discuss the clinical challenges, role of molecular profiling and review reported cases of pediatric thoracic LGFMSs.

## Introduction

Primary thoracic tumors are infrequent in children (1). Not only do clinical features often mimic more common non-oncologic conditions and medical emergencies may also arise due to mediastinal syndrome, thereby introducing additional diagnostic and therapeutic challenges. Depending on patient age and tissue of origin, neoplastic differentials encompass pleuropulmonary blastoma (PPB), inflammatory myofibroblastic tumor, germ cell tumor, lymphoid proliferation, Ewing sarcoma and other soft-tissue sarcoma. Low-grade fibromyxoid sarcoma (LGFMS) is a rare, locally aggressive malignancy that originates mainly from the proximal extremities with a median age of diagnosis of 33 years (2). Herein, we present the clinical, histologic, and molecular features for a pediatric patient diagnosed with a primary thoracic LGFMS presenting with mediastinal compression, who was successfully managed with a multidisciplinary approach and extracorporeal membrane oxygenation (ECMO) support.

## Case Description

**FIGURE 1:**
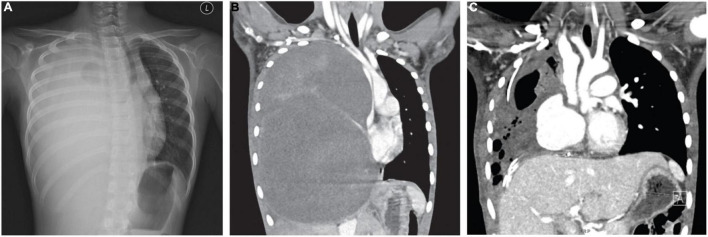
**(A)** CXR and **(B)** CT revealed a giant mass of 16.9 cm × 15.2 cm × 22.4 cm (AP × W × H) expanding the right thorax with significant compression on the mediastinum. **(C)** Post-operative CT indicates partial re-expansion of the right lung.

A previously healthy nine-year-old girl presented with six months of cough, weight loss, and recent onset of dyspnea. Examination showed reduced air entry over the right lung, tracheal deviation to the left and facial puffiness, suggestive of superior vena cava syndrome. Subsequent X-ray and computed tomography (CT) confirmed a solid mass lesion occupying and expanding the entire right hemithorax, measuring 17 × 15 × 22 cm (Figure 1). There was total atelectasis of the right lung, expansile remodeling of the rib cage, thoracic scoliosis concaving to the right, and leftward displacement of mediastinal structure including the trachea and narrowing of the superior vena cava.

Given these imaging features, Type III PPB was initially considered, with differentials including inflammatory myofibroblastic tumors and other soft-tissue sarcomas. With such extent of disease, the patient was considered to be at an extremely high risk for anesthetic procedures, core biopsy was thus done under local anesthesia, mitigating the concerns associated with use of systemic agents. Frozen section indicated the presence of spindle cells. In view of the life-threatening picture, empirical treatment for PPB according to the IVADo regimen (ifosfamide, vincristine, doxorubicin, actinomycin) was started and the patient was monitored closely in the intensive care unit (3).

**FIGURE 2:**
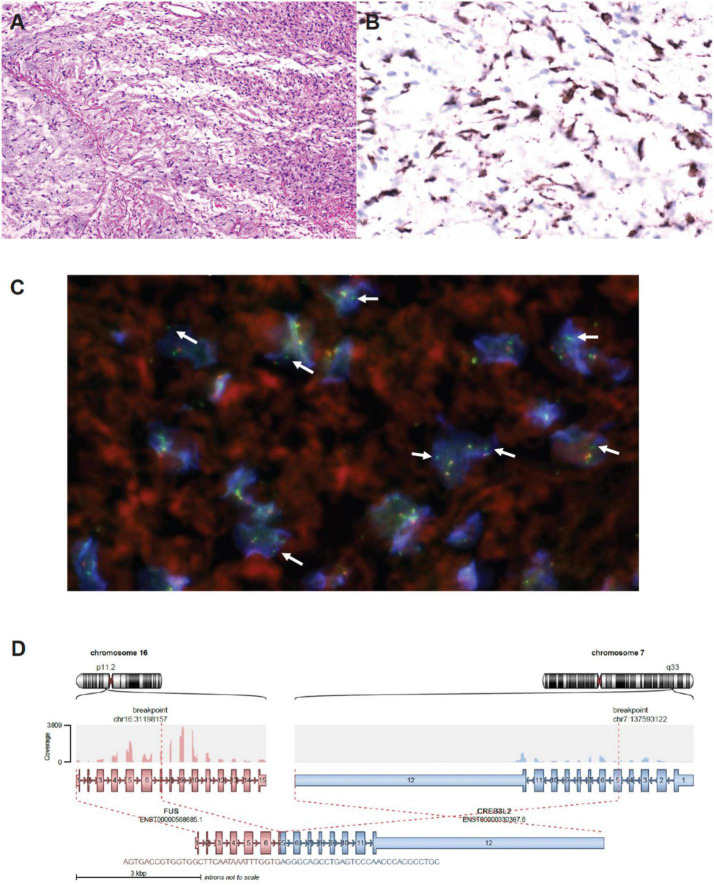
**(A)** H&E staining on the tumor biopsy specimen – abnormal spindle cells with scattered small-sized blood vessels; focal myxoid area was noted without mitosis, necrosis or rosette formation. **(B)** Diffuse and strong MUC4 positive shown in the spindle cells. **(C)** FISH study with nuclei showing isolated green FISH signal (5′end of *FUS* gene) compatible with presence of *FUS* translocation (courtesy of Prof Ka-Fai To, Chinese University of Hong Kong). **(D)** RNA sequencing confirming *FUS*-*CREB3L2* chimeric transcript.

**FIGURE 3:**
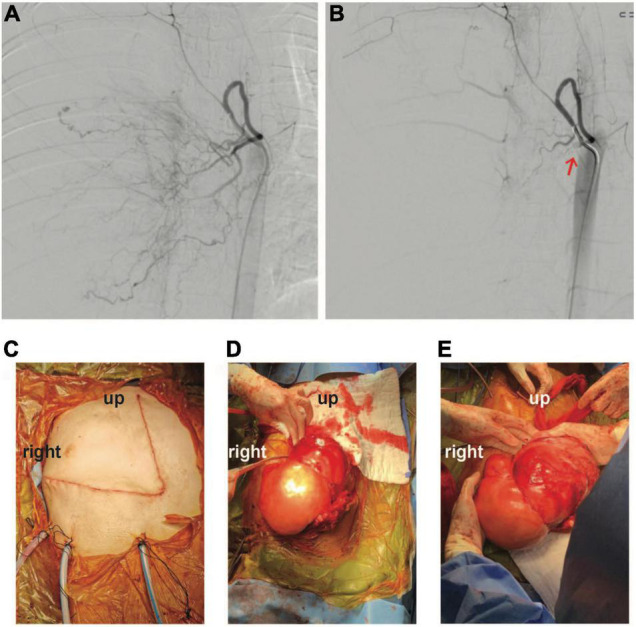
Angiographic findings **(A)** before and **(B)** after embolization of tumor feeding vessels (coil indicated by red-arrow). **(C–E)** Intra-operative photo illustrating successful en bloc excision of tumor through a hemi-clamshell incision.

Nonetheless, reassessment CT two weeks later showed no tumor shrinkage. Histological analysis on fixed tissue obtained from core biopsy indicated abnormal spindle cells with focal myxoid areas, and absence of mitosis, necrosis or rosette formation (Figure 2). On immunohistochemistry (IHC), MUC4 positivity was detected, and fluorescence *in situ* hybridization study indicated the presence of *FUS* translocation, suggesting LGFMS (4). RNA-sequencing further confirmed the presence of *FUS*-*CREB3L2* fusion transcript. Armed with the understanding that LGFMS is unresponsive to chemotherapy or radiotherapy, definitive surgery with a multidisciplinary team approach was undertaken one month after diagnosis. The patient had pre-operative cannulation of femoral vessels with ECMO standby and embolization of feeding vessels from the right bronchial artery (Figure 3). Endotracheal intubation was achieved without complication; medial sternotomy with right anterolateral thoracotomy (hemi-clamshell approach indicated without the need for supraclavicular incision in view of the lack of blood supply from the head and neck vessels) was then performed, followed by a successful en bloc resection of the encapsulated tumor attached to the pericardium. Intra-operatively the patient developed supraventricular tachycardia requiring cardioversion with brief internal cardiac massage. In view of borderline hemodynamic status and impaired right ventricle dysfunction during wound closure, peripheral veno-arterial ECMO (VA-ECMO) support was established. The resected specimen measured 27 × 21 × 15 cm and weighed 2793 g.

Post-operatively, the patient was maintained on VA-ECMO for two more days due to pulmonary hypertension. She was kept on ventilator for total of 6 days; her mediastinal drain was kept for 4 days, urinary catheter for 5 days and chest drains for 13 days. CT thorax with angiogram 3 weeks after surgery shown a hemodynamically insignificant segmental pulmonary embolism, which was managed with enoxaparin. Patient recovery was otherwise uneventful, with normal ventricular function as assessed by echocardiography. She was put on nocturnal oxygen 0.5 L/min for 9 months, and received intensive rehabilitation with joint input from our pediatric respirologists and physical therapists. Bimonthly serial lung function tests including lung volumes measurement and DLCO showed mild restrictive pattern. The patient had been monitored clinically and radiographically (with CT) every 4 monthly, remaining in remission thus far 12 months from diagnosis (Figure 1C). She was referred to the spine team for monitoring of mild scoliosis and had not required any interventions. Quality of life scores based on the PedsQL tool at 6 months from operation were 93 and 88% for psychosocial health as reported by parent and patient, respectively, and 100% in physical health by both the parent and patient.

## Discussion

First described in 1987, LGFMS is predominantly an adult-onset condition with less than 20% of patients presenting at less than 18 years-of-age (2, 5–7). Despite its bland histologic appearance and indolent course, the tumor remains locally aggressive and carries a small risk of distant metastasis (5). Tumor may progress over the course of decades (8). Wide local excision remains the standard-of-care, with local and distant failure rates of 9 and 6%, respectively, although these figures might be underestimations due to the propensity for late recurrence (9, 10). The pathognomonic molecular driver for LGFMS was determined to be fusion between *FUS* and *CREB3L2* (95%) or *CREB3L1* (5%), while MUC4 expression represents a sensitive and specific surrogate marker on IHC studies (11–13). At the epigenomic level, LGFMS remains a molecularly distinct entity from other soft-tissue sarcomas including sclerosing epithelioid fibrosarcoma, which shares histologic similarities with LGFMS but is driven instead by *EWSR1-CREB3L1/2* fusions (14). The systematic incorporation of molecular studies in the work-up of soft-tissue sarcoma has become indispensable for the prompt and precise classification of tumor type.

Whilst pulmonary metastasis is the commonest mode of distant spread, primary intra-thoracic LGFMS have only been anecdotally reported (7, 15–21). These unusual lesions have been described to arise from the pleura, lung parenchyma, and mediastinum. In our patient, the intraoperative findings suggested a pericardial origin of the tumor. In the pediatric age group, Steiner and colleagues reported on a 12-year old girl with a 23 cm intrathoracic LGFMS that appeared to arise from the posterior thoracic wall without mediastinum invasion, was surgically treated uneventfully (19), whereas a 15-year old boy with a 3 cm thoracic lesion was described as part of the French Sarcoma Group experience (5). As part of Children’s Oncology Group ARST0332 cohort of non-rhabdomyosarcoma soft-tissue sarcoma, one 12-year old boy with intrapulmonary LGFMS without mass effect shown in axial CT thorax was reported (7). Our patient likely represents the youngest reported individual with thoracic LGFMS in the literature. Of note, the splaying of ribs and scoliosis present in our patient are evidence for chronicity suggesting an even earlier onset of our patient’s tumor.

Mediastinal mass syndrome (MMS) as a result of neoplastic growth represents an oncologic emergency, which can manifest as life-threatening cardiovascular decompensation as a result of mass effects on the surrounding anatomical structures (22, 23). According to previous publications, patients have higher risk of acute cardiovascular decompensation if they present with orthopnea, stridor, wheeze, shortness of breath, syncope, and upper body edema; most of which were present in our patient (24, 25). Radiological assessment including CT thorax and echocardiogram is recommended to facilitate risk assessment prior to sedation and interventions. Prior to any treatment, it is important to maintain spontaneous respiration, avoid sedation, avoid neuromuscular blocking agents, and preload augmentation by optimizing the fluid status and pharmacologic support (24). Corticosteroid is of value mainly in lymphoid malignancies although close monitoring for resultant tumor lysis syndrome is essential. Timely transferal to a tertiary referral center with experience in cardiothoracic surgery and mechanical circulatory support is key in preventing catastrophic cardiorespiratory decompensation. Our case also demonstrated that ECMO can be successfully used in pediatric patients with massive mediastinal mass as a cardiovascular support modality during surgical treatment, for which there is only a limited number of pediatric case reports (26, 27). Multi-disciplinary input is essential to facilitate tumor resection and to minimize morbidity and mortality. Beyond the acute phase, pulmonary rehabilitation is key to ensure the patient’s long-term well-being and functional outcome, alongside radiographic surveillance which remains essential in order to monitor potential disease recurrence.

## Conclusion

We present the clinical course and molecular finding of a rare case of pediatric thoracic LGFMS. Molecular profiling facilitates the classification and management of uncommon soft-tissue tumors while pre-emptive multi-modal measures are integral to the successful management of patients with mediastinal syndrome.

## Data Availability Statement

The datasets for this article are not publicly available due to concerns regarding participant/patient anonymity. Requests to access the datasets should be directed to the corresponding author.

## Ethics Statement

Ethical review and approval was not required for the study on human participants in accordance with the local legislation and institutional requirements. Written informed consent from the participants’ legal guardian/next of kin was not required to participate in this study in accordance with the national legislation and the institutional requirements. Written informed consent was obtained from the minor(s)’ legal guardian/next of kin for the publication of any potentially identifiable images or data included in this article.

## Author Contributions

YC, DK, and AL: conceptualizing and initial drafting. YC, AK, LY, and AL: data collection. DK and AL: supervision. All authors contributed to the patient care, critical review, and final approval.

## Conflict of Interest

The authors declare that the research was conducted in the absence of any commercial or financial relationships that could be construed as a potential conflict of interest.

## Publisher’s Note

All claims expressed in this article are solely those of the authors and do not necessarily represent those of their affiliated organizations, or those of the publisher, the editors and the reviewers. Any product that may be evaluated in this article, or claim that may be made by its manufacturer, is not guaranteed or endorsed by the publisher.
